# Breastfeeding Rates and Growth Charts—the Zhejiang Infant Feeding Trial

**DOI:** 10.3390/ijerph120707337

**Published:** 2015-06-30

**Authors:** Bingquan Zhu, Jian Zhang, Liqian Qiu, Colin Binns, Jie Shao, Yun Zhao, Zhengyan Zhao

**Affiliations:** 1 Children’s Hospital, Zhejiang University, Hangzhou, Zhejiang 310006 China; E-Mails: ispring2003@ 163.com (B.Z.); zhangj@zju.edu.cn (J.Z.); shaojie@zju.edu.cn (J.S.); 2 Women’s Hospital, School of Medicine, Zhejiang University, Hangzhou, Zhejiang 310006, China; E-Mail: qiulq@zju.edu.cn; 3 School of Public Health, Curtin University, Perth 6102, Australia; E-Mail: Y.Zhao@exchange.curtin.edu.au

**Keywords:** growth chart, mother’s perceptions, percentiles, breastfeeding rates, China

## Abstract

A randomised control trial was undertaken in Hangzhou, China, to study the influence of the growth chart used on breastfeeding rates. Mothers with infants who were being fully breastfed at 6 weeks after birth (n = 1602) were invited to participate in the trial; 1415 agreed to participate and 1295 completed the study. Two growth charts were used, one that was heavier for the first six months of life (Chart A, n = 686) and a lighter growth chart (Chart B, n = 609). Mothers were interviewed and infants measured at 6 weeks and 3, 4, 5 and 6 months after delivery. At 6 months the full breastfeeding rates were 18.1% in the group using the heavier growth chart compared to 22.8% in the lighter growth chart group. After adjusting for potential confounders this difference remained significant (aOR 1.41, 95% confidence intervals 1.02, 1.93). These results suggest that breastfeeding rates may be influenced by the type of growth chart used. Mothers who perceive that their infants are not growing adequately (*i.e.*, using the heavier charts) may introduce other foods to their infants earlier than mothers using the lighter chart. While a larger trial is required to confirm the results, in the interim it is suggested that if heavier growth charts are used, a lower percentile line could be used to assess adequacy of growth.

## 1. Introduction

The most practical measures of nutritional status in childhood are comparisons with growth reference charts, most commonly showing weight for age, height for age and weight for height. Monitoring of growth is widely practiced in maternal and child health clinics where infants are measured, vaccinations are given and the mothers receive postnatal care and health education. For practical reasons growth monitoring usually involves only weight for age at approximately monthly intervals up to six months of age and then less frequently until five years of age. Health professionals and parents use growth charts, preferably following a trend over several months, to assess adequacy of infant feeding. Exclusive breastfeeding for six months, followed by continuing breastfeeding, is the recommended and best way to feed all infants and is supported by most health authorities [1,2,3]. Breastfeeding has many benefits for children and their mothers [4,5,6].

Regular measurement and plotting against a growth reference is an important monitoring tool as parents often do not recognise the rate of growth or being overweight in themselves or their children [7,8]. Together with vaccination it is probably the most common intervention in pediatrics and family medicine. The term “growth reference” refers to the set of data used to compile a growth chart. Reference implies that the growth chart is used to plot the individual child’s growth over time and the trend in growth is more important than the absolute position on the chart. In 1978 the World Health Organization adopted the growth reference produced by the US National Centre for Health Statistics for international use [9,10]. These charts were included in the personal health records of many countries for use by parents and health workers as an ongoing record of a child’s growth and development. In 2000 the US charts were revised to eliminate some minor anomalies around two years of age [11]. In particular, the data used for infants was updated, the calculation of some percentiles was revised and this version became the most commonly used growth reference worldwide.

However it was widely recognized that the U.S. growth data was not representative of children internationally and that exclusively breastfed babies grow at a slightly lower rate than the old WHO (NCHS) growth reference [12,13]. In 1997 de Onis expressed concerns about the existing growth reference: “the NCHS curves are inappropriate for healthy, breastfed infants. Recent research shows that infants fed according to recommendations by the WHO and who live under conditions that favor the achievement of genetic growth potentials grow less rapidly than, and deviate significantly from, the NCHS reference” [12]. It was thought at the time that the heavier growth reference might lead health professionals to ^“^make faulty decisions regarding the adequate growth of breastfed infants, and thus to mistakenly advise mothers to supplement unnecessarily or even to stop breastfeeding altogether” [12]. Early inappropriate termination of breastfeeding is an important public health problem leading to increased health problems and costs. “The premature introduction of complementary foods can have life-threatening consequences for young infants in many settings, especially where breastfeeding's role in preventing severe infectious morbidity is crucial to child survival” [12].

These concerns led to the preparation of a new growth reference by the WHO and the development process has been described in great detail [14,15]. Previously WHO has used the term “growth reference”, but now felt confident enough about the new growth study to refer to it as a “growth standard”. When compared to the older CDC 2000/WHO reference, the new 2006 WHO standard is heavier during the first six months of life [14,16,17,18]. A study of 9000 infants from three countries showed that using the WHO standard (compared to the older WHO/CDC reference) the number of infants under 6 months of age who were classified as undernourished was increased and for older infants the proportion of overweight was increased [19]. After the first six months of life the new WHO reference trends lower than the older CDC reference reflecting that fact that by 12 months breastfed infants are leaner than their formula fed infants[14]. The critical period for breastfeeding is the first six months of life when exclusive breastfeeding, or at least full breastfeeding, is important for health. If an increased proportion of infants are classified as underweight, concerned parents may stop exclusive breastfeeding and introduce complementary or supplementary feeds prematurely. Breastfeeding is unlike most health interventions, in that a decision to cease breastfeeding is final and cannot be reversed. The objective of this study was to evaluate the impact of the use of a heavier growth chart based on the new WHO growth charts, compared the previous CDC growth chart, on infant feeding practices in Hangzhou (Hangzhou, China), using a randomized controlled trial design. 

## 2. Methods and Materials 

In Zhejiang Province, Hangzhou, China, all mothers are required to deliver their infants in a hospital or a maternal and child health facility. All mothers are asked to bring their infants for examination and vaccinations at infant health clinics at regular intervals for the first year after birth and compliance rates in Hangzhou are very high. At the clinic, children are examined and measured and growth is recorded on the infant’s record, which includes a growth chart. A randomized controlled double-blind trial was undertaken to compare the effect on ‘full breastfeeding’ rates of the use of two growth charts, one with a higher (heavier) growth rate (Chart A) and another with a lower (lighter) growth rate (Chart B). Chart A was a unisex version of the new WHO growth chart and Chart B used the CDC growth reference. The trial was limited to the first six months of life as this is the most critical period for breastfeeding. Information on the study was given to mothers while they were in hospital and randomization to the different charts was done at the six weeks post-partum visit contact using a computer random number program. Growth charts were distributed to the mothers at this time and a duplicate chart was kept in the clinic records. The exclusion criteria were “not full breastfeeding at six weeks”, “multiple births”, “infants who had spent more than 4 days in neonatal intensive care units” and “mothers who were not capable of participating because of incapacity”. The growth charts were unisex in design, midway between centile lines for both genders and included the 5th, 50th and 95th percentiles. The difference between the 50th percentiles of the two charts was 0.4 kg at 4 months. The mothers and the health staff were both blinded to the charts being used. The Infant Health Records include information on the infants’ growth, health, hospital and clinic attendances and feeding patterns. During the study there were no changes in the way data was routinely collected from all mothers or the way routine child care and vaccinations were provided. In addition mothers were asked to complete an initial and a follow-up questionnaire. The questionnaires used in the study were a shortened version of the initial and follow up questionnaires from the Xinjiang and Zhejiang Infant Feeding Studies [20,21]. Both questionnaires have been extensively used in previous studies. The infants were followed up at 6 weeks and 3, 4, 5 and 6 months. The infant’s weight, height and feeding patterns were recorded on the growth chart and in the clinic records, and questionnaires completed at every visit. Mothers kept the growth chart in their care between visits, although duplicate records were also kept in the clinics. At every visit, the doctor explained the infant’s growth to the parents and gave routine health and nutrition advice as well as commencing standard vaccinations. If the infant’s weight was slowing or fell below the 5th percentile, the medical staff would enquire about any health or feeding problems. Staff were not given any additional instructions on advice to be given to parents, but were trained to emphasize the importance of full breastfeeding.

In China currently there is a high rate of prelacteal and early complementary feeding [22,23]. For this reason the endpoint of the trial was specified as “full breastfeeding” rather than “exclusive breastfeeding”. The operational definition of “full breastfeeding” used was “no feeds other than breastmilk in the past 24 hours” and it is recognized that this may be referred to as exclusive breastfeeding by other authors [24]. 

At the first clinic visit mothers were asked if they had read the information and they were asked to sign the consent form. They were informed that the data would be kept confidential and that only aggregated statistical information would be released. The study was approved by the Children’s Hospital of Zhejiang University Human Ethics Research Committee. We selected six hospitals located in urban areas of Zhejiang Province for the study. The sample size to be recruited was calculated on the basis of previous studies in Zhejiang. At 6 months the ‘any breastfeeding rate’ was estimated to be 62% based on our previous studies [20,25]. A sample of 600 mothers in each group would allow the detection of a difference of 5% in breastfeeding rates (Power 0.8, *p* = 0.95). Adjusted odds ratio of fully breastfeeding at 6 months comparing between Chart A (reference) and Chart B growth charts was assessed using multiple logistic regression analysis, controlling for possible confounders such as gender, premature delivery, birth weight, delivery way, mother age, educational level and family income and the random effect of the community health service centers. Analysis was undertaken using SPSS (V20) and binary logistic regression using SAS [26,27]. 

## 3. Results and Discussion

At birth 2995 mothers were given information about the growth study and invited to participate in the study and 396 (13.2%) declined to receive further information on the study. The most common reason given was difficulty in attending the hospital follow up clinics due to distance. At six weeks 1602 mothers were still ‘fully breastfeeding’ their infants and 1415 were included in the study. During the study a further 120 mothers who had moved to another location and could not be contacted were lost to follow-up, resulting in a sample of 1295 (80.8%) who were included in the analysis. This was not unexpected as there are an estimated 250 million rural-urban migrant workers in China and Zhejiang Province has a large mobile population. 

The final sample included 686 infants in the Chart A group and 609 infants in the Chart B group and the demographic characteristics of the sample are shown in Table 1. There were no differences between the two groups except for slightly higher incomes in families in the Chart B group and some differences in maternal employment. Previous studies in China had shown that higher income families in Hangzhou, the capital city of Zhejiang Province, were less likely to initiate breastfeeding and had a shorter duration of breastfeeding [20,25]. 

**Table 1:** Demographic characteristics of the two groups and univariate comparisons.

Variables	Chart A	Chart B	χ^2^ Test	
	Group n (%)	Group n（%)	χ^2^ Value	*p* Value
**Gender**				
Total	686 (53.0)	609 (47.0)	0.02	0.876
Boys	331 (48.5)	291 (48.1)		
Girls	351 (51.5)	314 (51.9)		
**Preterm**				
Yes	18 (2.6)	21 (3.5)	0.75	0.386
No	668 (97.4)	588 (96.5)		
**Birth weight**				
LBW (<2500 G)	34 (5.0)	18 (3.0)	3.35	0.067
Normal	628 (92.6)	556 (91.3)		
HBW (>4000 G)	24 (3.5)	35 (5.8)		
**Delivery method**				
Vaginal Delivery	240 (35.8)	194 (32.7)	1.30	0.254
Caesarean section	431 (64.2)	399 (67.3)		
**Mother’s age (years)**				
<25	182 (26.5)	141 (23.2)	3.23	0.357
25–30	364 (53.1)	331 (25.6)		
30–35	116 (16.9)	107 (17.6)		
≥35	24 (3.5)	30 (4.9)		
**Mothers employment**				
Manual Labor	109 (16.1)	69 (11.4)	12.2	0.032
Business	52 (7.7)	57 (9.4)		
Farmer	64 (9.4)	66 (10.9)		
Civil service	143 (21.1)	159 (26.2)		
Housewife	195 (28.8)	151 (24.9)		
Other	115 (17.0)	104 (17.2)		
**Education**				
Junior high or less	174 (25.9)	158 (26.3)	0.40	0.819
Senior high school	234 (34.8)	199 (33.1)		
College	265 (39.4)	244 (40.6)		
**Family income (10,000 RMB/year)**				
<3	128 (19.1)	118 (19.9)	11.41	0.010
3–6	309 (46.1)	239 (40.4)		
6–10	180 (26.9)	156 (26.4)		
≥10	53 (7.9)	79 (13.3)		

Notes: LBW = low birth weight (<2500 G) and HBW = high birth weight (>4000 G).

There were no differences in mean weight between the two groups at 6weeks and 3, 4, 5 and 6 months（p > 0.05）as shown in Table 2. The unadjusted results of the study are shown in Table 3 and the final multivariable model is presented in Table 4. At 6 months there was a difference of 4.7% in the prevalence of full breastfeeding between the two groups. The other significant factors were the mothers’ education and type of employment. After adjustment for covariates and random effects of the community health service centres, the adjusted odds ratio for ‘full breastfeeding’ at 6 months was 1.41 (Model 1: 95% CI (1.02, 1.93), Model 2: 95% CI (1.02, 1.94)) where the Chart A (the heavier chart) was the reference category, (see Table 4). At 6 months the prevalence of fully breastfed infants who were randomised to the lighter Chart B was 22.8% compared to 18.1% in Chart A. 

**Table 2:** Comparison of the weight-for-age of two groups (Mean, SD).

Chart	6 Weeks	3 Months	4 Months	5 Months	6 Months
Chart A group	4.99 (0.57)	6.70 (0.76)	7.41 (0.81)	7.99 (0.85)	8.46 (0.90)
n	741	732	708	687	686
Chart B group	4.97 (0.56)	6.65 (0.77)	7.38 (0.83)	7.95 (0.89)	8.39 (0.95)
n	674	631	619	612	609
P value	0.295	0.167	0.172	0.138	0.083

Note: Weight in kg.

**Table 3:** The demographic characteristics of the ‘full breastfeeding’ group and other infant feeding at 6 months.

Variable	Full Breastfeeding n（%）	Other Feeding n（%）	*χ*^2^ Test	
			*χ*^2^ Value	*p* Value
**Growth Chart**				
Chart A	124 (18.1)	562 (81.9)		
Chart B	139 (22.8)	470 (77.2)	4.206	0.040
Gender				
Boy	128 (20.4)	498 (79.6)	0.025	0.874	
Girl	133 (20.1)	534 (80.1)			
**Preterm**					
Yes	9 (23.1)	30 (76.9)	0.062	0.803	
No	254 (20.1)	1008 (79.9)			
**Birth weight**					
LBW (<2500 G)	8 (15.4)	44 (84.6)	0.78	0.676	
Normal	243 (20.4)	947 (79.6)			
HBW (>4000 G)	12 (20.3)	47 (79.7)			
**Delivery method**					
Vaginal	89 (20.5)	346 (79.5)	0.01	0.984	
Caesarean section	169 (65.5)	666 (65.8)			
**Mother’s age (years)**					
<25	61 (18.7)	265 (81.3)	0.72	0.866	
25–30	141 (20.2)	557 (79.8)			
30–35	49 (22)	174 (68)			
≥35	12 (22.2)	42 (77.8)			
**Mothers’ employment**				
Labourer	25 (14)	153 (86)	25.45	<0.001
Business	18 (16.2)	93 (83.8)		
Farmer	24 (28.4)	106 (71.6)		
Civil service	86 (15.8)	217 (84.2)		
Housewife	55 (15.8)	294 (84.2)		
Other	55 (25.1)	164 (74.9)		
**Education(mother)**				
Junior high or less	58 (17.5)	274 (82.5)	7.77	0.021
Senior high school	77 (17.7)	358 (82.3)		
College	123 (24)	390 (76)		
**Family income (10,000/year)**				
<3	55 (22.3)	192 (77.7)	0.76	0.859
3–6	109 (19.9)	440 (80.1)		
6–10	67 (19.7)	273 (80.3)		
≥10	26 (19.3)	106 (80.7)		

Notes: Family income is expressed in Chinese yuan (1USD = 6.08 yuan).

**Table 4:** Multivariable model of ‘full breastfeeding’ at 6 months by growth chart type.

Chart	OR	95% CI	*T* Value	*p* Value
Univariate analysis				
Chart A	1			
Chart B	1.39	1.02–1.91	2.07	0.039
Multiple analysis-1 **^a^**				
Chart A	1			
Chart B	1.41	1.02–1.93	2.11	0.035
Multiple analysis-2 **^b^**				
Chart A	1			
Chart B	1.41	1.02–1.94	2.10	0.036

Notes: **^a^** Model 1: The covariates included are gender, premature delivery, birth weight and delivery way. **^b^** Model 2: The covariates included are gender, premature delivery, birth weight, delivery way, mother age, educational level and family income; Chart A represents a growth trajectory that is heavier than Chart B in the first six months of life. Random effect was adjusted in both Model 1 and Model 2 to allow for the use of six outpatient clinics for follow-up.

The results of this study show that the use of a heavier growth chart can lead to a lower prevalence of “full breastfeeding”. Mothers who perceive that their infants are not growing adequately (*i.e.*, from using the heavier charts) may introduce other foods to their infants earlier than mothers using the lighter chart. The new WHO growth charts are technically superior to previous growth references, have been widely promoted by the WHO and are now being introduced in many countries. The growth curve that they present is more physiological than older references. However the fact that the new WHO reference is heavier in the first six months has raised concerns about possible impacts on breastfeeding rates [28]. In this study we found the weight-for-age of two groups were same at 6 weeks and 3, 4, 5 and 6 months, but “full breastfeeding” rates are lower in the heavier growth chart group. 

Suboptimal breastfeeding is estimated to be responsible for approximately 1.4 million child deaths annually and three quarters of these are probably due to non-exclusive breastfeeding [29,30]. It appears that when mothers and health workers see their infant’s growth slipping below percentile lines on the chart, they are inclined to introduce formula or complementary foods or even stop breastfeeding altogether. The early discontinuation of exclusive breastfeeding (before six months) has implications for public health [3,31]. Bhutta has emphasised the importance of exclusive breastfeeding by noting that the promotion of breastfeeding could lead to a 11.6% reduction in the number of infant deaths [32]. A reduction in breastfeeding rates could also influence health outcomes and have cost implications for the provision of health services with an increase in admissions from diarrhoea, lower respiratory tract infections and other conditions. There are also considerable cost savings for mothers who continue to breastfeed. In the longer term breastfeeding also reduces chronic disease in later life with considerable public health benefit [2,33]. The slight difference in incomes and birth weight between the two groups would reduce the power of the study, but had no impact on the outcome, when their effects were adjusted for as covariates in the logistic regression model.

The new WHO growth standard has now been introduced in many countries. It might have been preferable if the new WHO growth charts had been the subject of a randomized controlled trial in a similar way to other important health interventions. A cross over study of health workers in Malawi found that the use of the new WHO chart (heavier chart) risked reducing the exclusive breastfeeding rate due to a perceived lower growth rate [34]. 

This study is the first reported trial of mothers, infants and health workers and the results of this study suggest that where the new growth charts are in use, education programs are needed for health workers and parents to ensure continued exclusive breastfeeding despite small deviations below percentile lines. All infant growth charts, at least those under the age of 12 months, should include a lower percentile (perhaps the 2nd percentile) as the lowest line on the growth chart. While the trajectory of growth remains most important in evaluating nutrition status and the adequacy of breastfeeding, the use of a lower percentile in heavier growth charts as an additional aid in determining growth adequacy could possibly reduce unnecessary intervention in breastfeeding infants. 

There are several limitations that need to be considered when interpreting the results of the study. This study used only two growth charts and was based on an urban sample. A larger study is required that includes both urban and rural areas in China that uses gender specific growth charts to confirm the results of this study, and to determine whether this difference in exclusive or full breastfeeding rates has clinical significance clinical significance.

## 4. Conclusions 

As far as we are aware this is the first randomized controlled trial of different growth charts to be published from Asia. The study showed that the use of a different (heavier) growth chart resulted in a small reduction in “full breastfeeding” rates. The public health impact of a reduction of “full breastfeeding” rates needs to be further assessed. However the study confirms the need to educate health workers on the correct use of growth charts to maximize the number of infants who are breastfed according to the WHO Recommendations [35]. This is particularly important if changes are made to the infant growth chart. The introduction of a new growth chart should include discussion of the most appropriate growth percentile to be used to assess breastfeeding and the implementation of education programs for health workers.
